# The Vacuole and Mitochondria Patch (vCLAMP) Protein Mcp1 Is Involved in Maintenance of Mitochondrial Function and Mitophagy in *Candida albicans*

**DOI:** 10.3389/fmicb.2021.633380

**Published:** 2021-02-04

**Authors:** Xiaolong Mao, Li Yang, Yiming Fan, Jiazhen Wang, Dongkai Cui, Dixiong Yu, Qilin Yu, Mingchun Li

**Affiliations:** Key Laboratory of Molecular Microbiology and Technology, Ministry of Education, College of Life Science, Nankai University, Tianjin, China

**Keywords:** *Candida albicans*, Mcp1, mitophagy, mitochondria, vCLAMP

## Abstract

The vacuole and mitochondria patches (vCLAMPs) are novel membrane contact sites in yeast. However, their role in autophagy has not been elucidated so far. In this article, the role of Mcp1, one core component of vCLAMP, in mitophagy of *Candida albicans* was investigated. Deletion of *MCP1* led to abnormal accumulation of enlarged mitochondria and attenuated stability of mitochondrial DNA (mtDNA) in *C. albicans* when cultured in non-fermentable carbon sources. Furthermore, the *mcp1*Δ/Δ mutant exhibited defective growth and degradation of Csp37-GFP. These results indicate that Mcp1 plays a crucial role in mitophagy and maintenance of mitochondrial functions under the non-fermentable condition. Interestingly, this deletion had no impact on degradation of Atg8 (the macroautophagy reporter) and Lap41 (the cytoplasm-to-vacuole targeting pathway marker) under SD-N medium. Moreover, deletion of *MCP1* inhibited filamentous growth and impaired virulence of the pathogen. This study provides an insight to vCLAMPs in cellular functions and pathogenicity in *C. albicans*.

## Introduction

*C. albicans* is one of the most common opportunistic pathogenic fungi in clinic (Kim and Sudbery, 2011). When invading the host cells, the primary stress that *C. albicans* encounters is environmental pressure like starvation. However, *C. albicans* responds to this pressure through autophagy (Palmer, 2008). Autophagy is a conservative degradation pathway of intracellular material. During the process of autophagy, the damaged proteins or organelles were wrapped by a double membrane, degraded in vacuoles or lysis, and the decomposition products return to the cytoplasm to participate in other biochemical reactions (Li and Vierstra, 2012). There are two types of autophagy, non-selective autophagy and selective autophagy. In yeast, selective autophagy is further divided into several kinds of pathways, e.g., Cvt pathway (cytoplasm to vacuole targeting), mitochondrial autophagy and peroxisome autophagy (Li and Vierstra, 2012; Delorme-Axford et al., 2015). Mitophagy is the selective autophagy, which can be activated by nutrient depletion or mitochondrial damage. The damaged mitochondria produce toxic by-products, e.g., reactive oxygen species (ROS), which can lead to cell death. Some studies have found that a complex connecting mitochondria and the endo-plasmic reticulum (ERMES) is an important factor in the process of mitophagy. Deletion of Mmm1 (a protein participating in ERMES) interferes mitophagy in *Saccharomyces cerevisiae* (Böckler and Westermann, 2014). However, as a bypass of ERMES, there is no report on the correlation between vCLAMP and autophagy (Elbaz-Alon et al., 2014; Ungermann, 2015; González Montoro et al., 2018).

The vacuole and mitochondria patch (vCLAMP) is a hot topic in fungal biology. In *Saccharomyces cerevisiae*, the Mcp1 (mitochondrial outer membrane protein) recruits Vps13 to mitochondria to form vacuole-mitochondria contacts (John Peter et al., 2017). Overexpression of *MCP1* can effectively compensate the damage caused by ERMES to mitochondria. Therefore we speculated that the vCLAMP core protein Mcp1 participates in autophagy.

External pressure from environment can damage mitochondria. Impaired mitochondria must be removed timely; otherwise it may cause further damage to cells (Kanki et al., 2011). Mitophagy is an important pathway for selective autophagy to eliminate mitochondria (Böckler and Westermann, 2014), which also has multiple physiological functions, relating to the maturation of erythrocytes, cell survival with injured mitochondria and neurodegenerative diseases like Parkinson’s disease (Bento et al., 2016; Gómez-Suaga et al., 2018). Carbon deficiency or breath inhibition can induce mitophagy. This article shows that the *mcp1*Δ/Δ mutant has decreased mitochondrial membrane potential (MMP) and self-stability in SC-G medium (non-fermentable carbon sources). Further investigations indicate that the mitophagy pathway and fusion of autophagosome with vacuole of *mcp1*Δ/Δ mutant are significantly suppressed. However, the macroautophagy and Cvt pathway are normal when cultured in SD-N medium (nitrogen starvation medium). Moreover, deletion of *MCP1* leads to deficiency in pathogenicity. These findings will enrich the cognition of vCLAMP in *C. albicans*.

## Materials and Methods

### Strains and Growth Conditions

All strains used in this paper were listed in Supplementary Table S1. The primer was listed in Supplementary Table S2. BWP17 was used as the wild-typle (WT) in the functional analysis and the parental strain for gene disruption. The *MCP1* gene knockout is performed by homologous recombination (Liang et al., 2018). The *MCP1:ARG4* fragment was transformed into the wild strain (WT). The heterozygous mutants (*mcp1:ARG4/MCP1*) were identified by PCR (*MCP1*-5det and *MCP1*-3det). After the *MCP1:URA3* fragment was transformed into the heterozygous mutant (*mcp1:ARG4/MCP1*) homozygous mutant strains (*mcp1:ARG4/mcp1:URA3*) were identified by PCR. The *URA3* was deleted in *mcp1*Δ/Δ (*URA3*) mutant strain to eliminate the interference of *URA3* to the experiment, confirmed by PCR. For systemic infection assays, the WT, *mcp1*Δ/Δ and *MCP1c* were transformed with *Pst*I/*Bgl*II-digested pLUBP (encoding *URA3* and its adjacent gene, *IRO1*), obtaining the *URA3*-reconstituted strains WT^*a*^, *mcp1*Δ/Δ^*a*^, and *MCP1*c^*a*^.

Cells were cultivated to log phase in YPD medium (2% glucose, 2% peptone, 0.5% yeast extract adding 80 μg/mL uridine). After collected, cells were transferred to SC-G medium (0.01% glucose, 0.67% yeast nitrogen base without amino acid, 0.2% amino acid drop-out mixture, 3% glycerin) and SD-N medium (1% glucose, 0.052% KCl, 0.152% KH_2_PO_4_, 0.052% MgSO_4_$7H_2_O supplemented with 0.1% traceelements, 0.1% vitamins) (Cui et al., 2019).

### Fluorescence Microscope

Fluorescence microscope was used to observe the location of proteins (Mcp1-GFP, Atg8-GFP, and Csp37-GFP). The strains were cultured in defined medium (SC-G and SD-N) at special time, and harvested. The green fluorescence was observed by fluorescence microscope (BX53, Olympus, Japan). After FM4-64 (dissolved in dimethyl sulfoxide (DMSO), final concentration 50 μg/ml, BBI) ((N-(3-triethylammoniumpropyl)-4-(6-(4-(diethylamino) phenyl) hexatrienyl) pyridinium dibromide)) staining at 30°C for 10 min (Fischer-Parton et al., 2000). The samples were observed by fluorescence microscope (BX53, Olympus, Japan). The morphology of mitochondria was observed after MitoTracker Red (dissolved in dimethyl sulfoxide (DMSO), final concentration 1 mmol/ml) staining at 37°C for 30 min (BX53, Olympus, Japan).

### Non-fermentable Carbon Sources Sensitivity Assay

To investigate the sensitivity of *mcp1*Δ/Δ to non-fermentable carbon sources (Kundu, 2011), cells were cultured overnight, washed twice by non-fermentable carbon sources medium (SC-G), transferred to SC-G to OD6_00_ of 0.1 and grown at 30°C with constant shaking at 160 rpm for 1–7 days. At the special time the same amount was taken on YPD solid plates and single colonies of *C. albicans* was counted. The data were shown from three replicates.

### Determination of Damaged Cell

The cells were cultured in YPD medium for 4–6 h, transferred to SC-G medium for 1 day. After propidium iodide (PI) (final concentration 5μg/ml) staining at 30°C for 10 min (Ratnakumar and Tunnacliffe, 2006), the samples were observed by a microscope (BX53, Olympus, Japan).

### Determination of Intracellular ATP, Reactive Oxygen Species (ROS), SOD, and CAT Levels

The intracellular ATP concentration was measured using the ATP-based creatine kinase catalyzing adenosine triphosphate and creatine to produce creatine phosphate. In ATP assay, cell numbers are calculated by OD_600_ where dead cells are excluded through PI-staining (Dong et al., 2015).

DCFH-DA (2′,7′-Dichlorodihydrofluorescein diacetate) was used to detect the total reactive oxygen species (ROS) concentration. The cells were cultured in SC-G at the special time, harvested and stained by DCFH-DA (final concentration 10 μmol/ml) at 37°C for 30 min. The samples were observed by a microscope (Ex = 480 nm, Em = 520 nm) (BX53, Olympus, Japan) (Yu et al., 2016).

The activity of SOD of cell was detected using a superoxide dismutase (SOD) assay kit (Hydroxylamine method) (Nanjing Jiancheng Bio., China). The samples were detected by a spectrophotometer (BIO-RED, United States) at OD_550_ nm (Li et al., 2013).

The activity of CAT was detected using the UV absorption method. The reaction solutions include protein extract and 100 mmol/L H_2_O_2_ and were detected by a spectrophotometer (OD_240_) (BIO-RED, United States).

### Immunoelectron Microscopy

The cells were washed twice by PBS, suspended in 2.5% glutaraldehyde fixative and fixed at 4°C for 12 h. Then cells were collected by centrifugation, 1% osmic acid was added, and the cells were suspended and fixed at room temperature for 1 h. Samples were fixed by gradient ethanol dehydration. The embedded samples were cut into 50–100 nm tissue sections using an ultra-microtome. Immunolabeling was performed using anti-GFP antibody (MBL598, 1:100) during 2 h at room temperature followed by Goat Anti-Rabbit IgG/Gold (10 nm) (Solarbio, 1:100 dilution) for 30 min at room temperature. The sample was observed by a transmission electron microscope (Tecnai G2 F-20, FEI, United States) (Elbaz-Alon et al., 2014).

### Immunoblotting

Samples were cultured in defined medium at special time. The cells extracts were subjected to SDS-PAGE and western blotting. The specific antibody, anti-GFP (MBL598), anti-Tubulin (MBL PM054), anti-Rabbit IgG (PROMEGA W4018) was used to enhance the luminescence intensity of the substrate. The protein imprint was detected by Western Blotting exposure meter (Tanon, 5200, multi).

### Measurement of Mitochondrial Membrane Potential (MMP)

After cells were cultured in SC-G at the special time, harvested and stained by JC-1 (dissolved in DMSO, final concentration 1 μg/mL, Sigma, United States) at 37°C for 30 min (Ratnakumar and Tunnacliffe, 2006; Pina-Vaz and Rodrigues, 2010; Dong et al., 2015; Liang et al., 2018; Lee et al., 2019). Samples were measured by flow cytometry (FACS Calibar flow cytometry, BD, United States). Gated region R1 (Ex = 488 nm, Em = 525 ± 20 nm) includes cells with intact mitochondrial membranes and gated region R2 (Ex = 514 nm, Em = 585 ± 20 nm) depicts cells with decreased of mitochondrial membrane potential. All samples were obtained from three independent experiments.

### Hyphal Development

Strains were cultured on the surface of solid hypha-inducing media (i.e., Spider and RPMI-1640 medium) at 30 and 37°C for 6 days. The strains were also culture in liquid hypha-inducing medium, i.e., the liquid RPMI-1640 medium, stained by Calcofluor White (CFW) (final concentration 10 mg/ml). The length of the hypha is measured by Image J.

### Virulence Assay

All procedures were performed in accordance with the “Guide to the Care and Use of NIH Laboratory Animals” and approved by the Ethics Committee of Nankai University (20160004). All mice used in this study were placed at a constant temperature of 24 ± 2°C in room, a 12 h light/dark cycle, lighted at 7:00 in the morning, and fed at conditions of freedom in Nankai University Medical College. Every effort was made to minimize animal suffering and animal populations.

Thirty ICR female mice (4 weeks old) were divided into 3 groups, and kept in the breeding room for about 1 week. The WT^*a*^, *mcp1*Δ/Δ^*a*^, and *MCP1c* ^*a*^ strains were cultured in YPD medium to 12 h, washed with PBS, and mice were injected through the tail vein (the injection cells were 5 × 10^6^). The death of the mice was observed and recorded every day.

In order to determine the load of *C. albicans* in the kidneys, after injection on the 3rd day mice in each group were sacrificed. The kidneys were dissected and weighed. One kidney was made into tissue sections and observed by a light microscope (BX53, Olympus, Japan), another one was fully ground and spread on YPD solid medium to calculate the number of colonies per gram of sample.

## Results

### Mcp1 Mainly Accumulated at the Mitochondrion-Vacuole Connecting Sites

**FIGURE 1:**
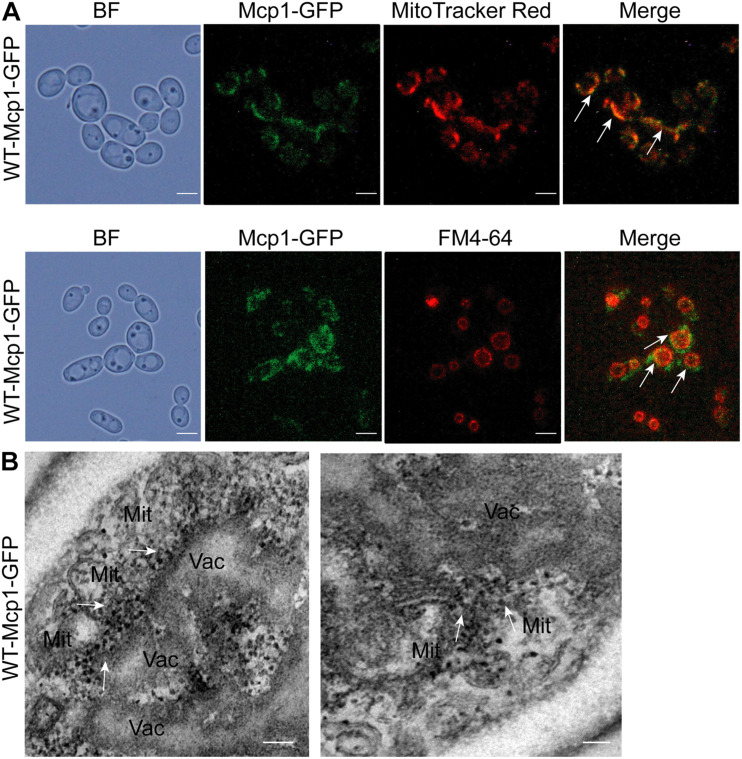
Intracellular localization of Mcp1 in *C. albicans*. **(A)** After stained by FM4-64 (indicating vacuoles) and MitoTracker Red (indicating mitochondria), the cells were observed by a fluorescence microscopy. Bar = 5 μm. **(B)** Subcellular localization of Mcp1-GFP. The Mcp1-GFP was visualized using Anti-GFP IgG/Rabbit and Goat Anti-Rabbit IgG/Gold (10 nm). The fusion protein accumulated in vCLAMP. Mit, mitochondria; Vac, vauoles. Bar = 200 nm.

The proteins of vCLAMP are located at the mitochondrion-vacuole connecting sites (John Peter et al., 2017). In the study, Mcp1 (orf 19.6550), a homologous protein of *S. cerevisiae*, was found through the online NCBI BLASTP software^1^ in *C. albicans*. To prove whether Mcp1 is a core vCLAMP protein, the location of Mcp1 in *C. albicans* was investigated by fluorescence microscope. The results showed that Mcp1-GFP was located both on the mitochondria stained by MitoTracker Red, and on the vacuolar membrane stained by FM4-64 (Figure 1A). Strikingly, overexpression of Mcp1 caused a massive expansion of the contacts between vacuoles and mitochondria (Figure 1B). Moreover, immunoelectron microscopy (IEM) further confirmed that Mcp1 mainly accumulated at vCLAMP (Figure 1B). Therefore, Mcp1 may be a protein accumulated at vCLAMP.

### Deletion of *MCP1* Led to a Growth Defect Under the Non-fermentable Condition

**FIGURE 2:**
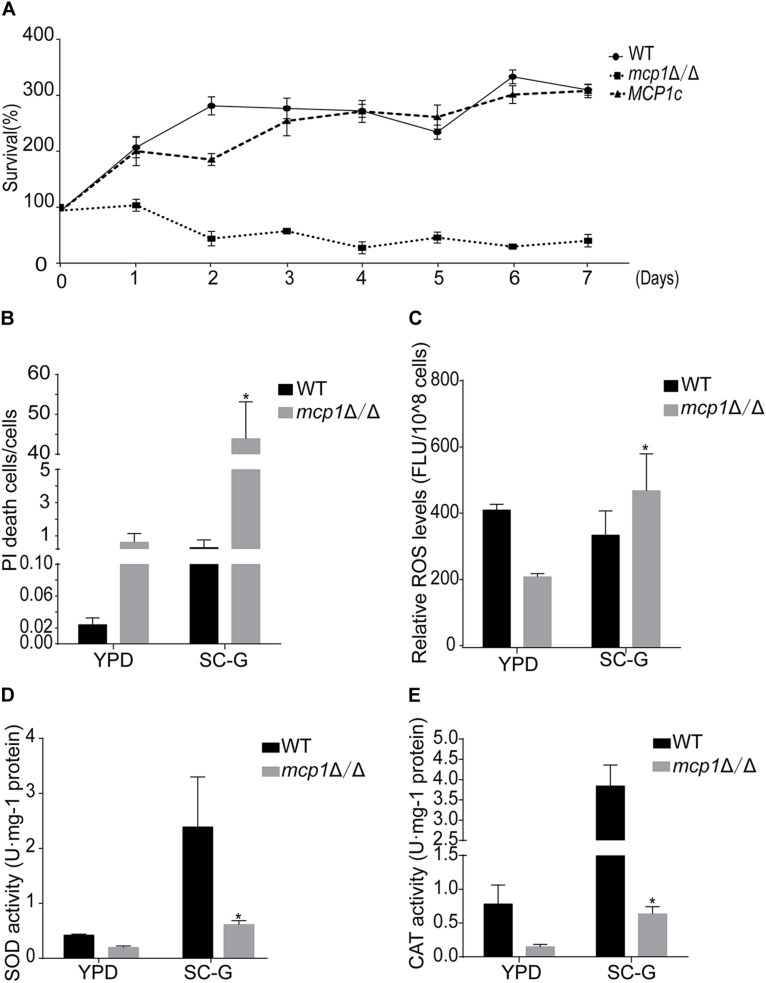
Deletion of *MCP1* impairs mitophagy in *C. albicans* in the SC-G medium. **(A)** Cell survival curves of the strains cultured in SC-G medium. The cells were cultured for 7 days. Colony forming unites (CFU) were measured to show the survival rate. The data were shown as means ± SD from three replicates. **(B)** Cell damage in the SC-G medium. The cells were observed by fluorescence microscope after PI staining. **(C)** Intracellular ROS levels revealed by DCFH-DA staining after cultured in SC-G for 1 day. **(D,E)** Activity of superoxide dismutase (SOD) and catalase (CAT) in the WT and mutant after cultured in SC-G medium for 1 day. *Indicates a significant difference between the mutant and the control strains (*P* < 0.05).

To find whether Mcp1 participates in mitophagy, the strains were cultured in SC-glycerol (SC-G) medium (the medium containing the non-fermentable carbon source glycerol) for further investigation. The outcome shows that deletion of *MCP1* gene impairs the growth on non-fermentable carbon sources (Figure 2A). Propidium iodide (PI) staining further showed that the mortality rate of *mcp1*Δ/Δ was significantly higher than the control strain (>40% vs. <1%) under SC-G medium (Figure 2B). Cell death is not only related to autophagy but also related to oxidative damage caused by reactive oxygen species (ROS) (Yu et al., 2016). To detect the effect of *MCP1* deletion on ROS, the ROS levels were tested after DCFH-DA (2′, 7′-dichlorodihydrofluorescein diacetate) stained. The test shows that the ROS level of the *mcp1*Δ/Δ mutant was significantly higher than the control strains in the SC-G medium (Figure 2C). Moreover, the activity of superoxide dismutase (SOD) and catalase (CAT) was decreased remarkably in *mcp1*Δ/Δ mutant (Figures 2D,E). These results proved that deletion of *MCP1* leads to cell death under the non-fermentable condition.

### Deletion of *MCP1* Disrupts Mitochondrial Function Under the Non-fermentable Condition

**FIGURE 3:**
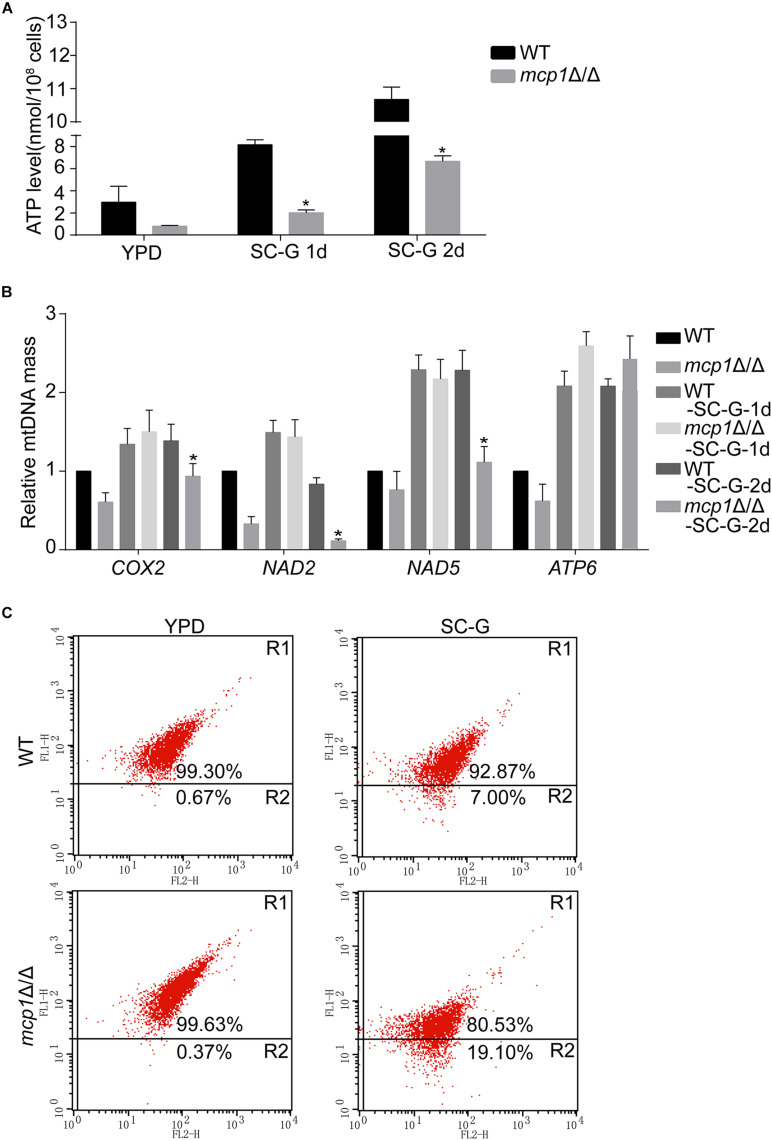
Loss of Mcp1 severely impairs mitochondrial function in SC-G medium. **(A)** Effect of *MCP1* deletion on ATP levels. *Significant differences between the mutant and the control strains (*P* < 0.05). **(B)** Effect of *MCP1* deletion on mtDNA quantity in SC-G medium. The mtDNA encoding genes *COX2*, *NAD2*, *NAD5*, and *ATP6* were analyzed by RT-PCR using *ACT1* as the normalization gene. *Indicates significant difference between the mutant and the control strains (*P* < 0.05). **(C)** Mitochondrial membrane potential revealed by JC-1 staining. The cells were cultured in SC-G medium. After incubated with JC-1 for 30 min at 37°C, samples were detected by flow cytometry. Gated region R1 shows the percentage of cells with intact mitochondrial membranes and gated region R2 shows the percentage of cells with loss of mitochondrial membrane potential.

Mitochondria are sack-like structures surrounded by two bilayers, which is indispensable for oxidative phosphorylation and ATP production (Gomes et al., 2011). In the test, the ATP level was decreased in the *mcp1*Δ/Δ mutant after cultured in SC-G medium for 1 day (Figure 3A). qRT-PCR analysis demonstrated that the expression of mitochondrial DNA (mtDNA, e.g., *COX2, NAD2, NAD5*, and *ATP6*) dropped in the same condition (Figure 3B; Dong et al., 2015).

Mitophagy maintains the function and number of mitochondria through removal of damaged or superfluous mitochondria (Kanki et al., 2011; Böckler and Westermann, 2014). In the experiment, the mitochondrial membrane potential (MMP) of *mcp1*Δ/Δ was decreased significantly on non-fermentable carbon sources (Figure 3C). The results suggest that Mcp1 plays an important role on maintaining the membrane potential. The findings suggest that deletion of *MCP1* severely impaired mitochondrial functions under the non-fermentable condition.

### Mitophagy Is Impaired in the *mcp1*Δ/Δ Mutant Under the Non-fermentable Condition

**FIGURE 4:**
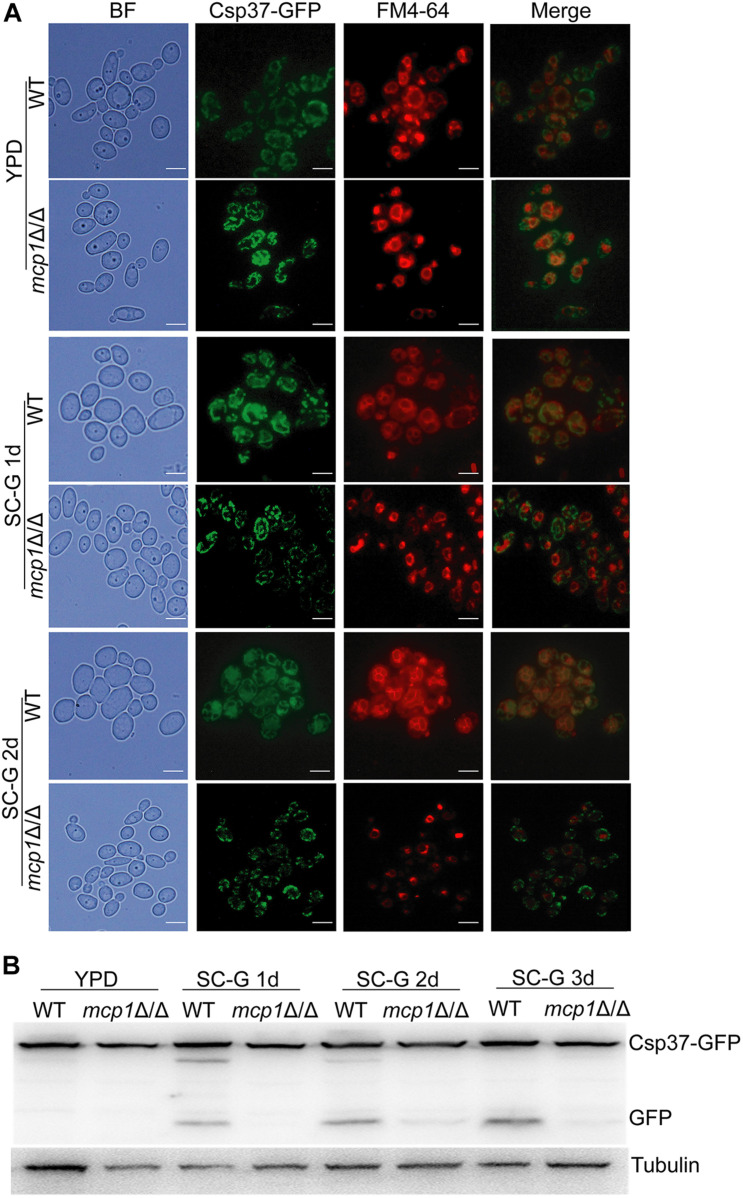
Deletion of *MCP1* results in abnormal localization and degradation of Csp37-GFP under the non-fermentable carbon source. **(A)** Deletion of *MCP1* leads to abnormal localization of Csp37-GFP. The cells were cultured in SC-G medium for 1 or 2 days. After stained by FM4-64, the cells were observed by fluorescence microscope. **(B)** Strains were cultured in SC-G medium for indicated times, and the total protein was extracted. Csp37-GFP and GFP were then detected by Western blotting using anti-GFP and anti-tubulin antibodies.

Mitophagy can be induced by non-fermentable carbon sources (Kundu, 2011). Csp37-GFP, a fusion protein localized on the outer membrane of mitochondria in *C. albicans* (Supplementary Figure S1), could be used as a mitophagy marker (Dong et al., 2015). During cultivation in the SC-G medium, Csp37-GFP was located inside the vacuoles in the WT (Figure 4A, up). However, Csp37-GFP was gathered around the vacuoles in *mcp1*Δ/Δ mutant (Figure 4A, bottom). Moreover, Western blotting revealed that the GFP alone was not detected in *mcp1*Δ/Δ mutant (Figure 4B and Supplementary Figures S2, S3).

**FIGURE 5:**
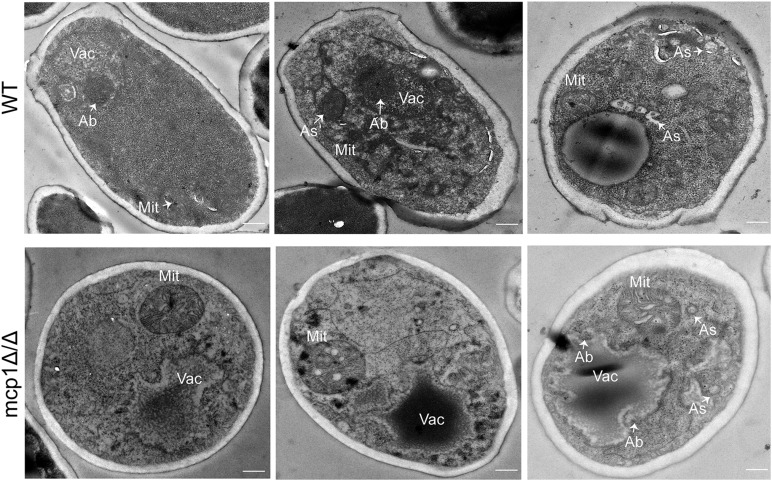
Deletion of *MCP1* caused the defect in autophagosome (As) formation in the SC-G medium. The strains were cultured in SC-G for 1 day, and observed by transmission electron microscopy (TEM). Bar = 500 nm. Vac, vacuole; Mit, mitochondria; As, autophagosome; Ab, autophagic bodies.

When mitophagy is activated, the double-membrane autophagosomes form and are transported from the cytoplasm to vacuole (Böckler and Westermann, 2014). As indicated by transmission electron microscopy (TEM), the WT cells formed normal autophagosomes in the cytoplasm under the non-fermentable condition (Figure 5, up). In contrast, the *mcp1*Δ/Δ mutant did not form any autophagosomes, and showed abnormally enlarged mitochondria (Figure 5, bottom). These results revealed that deletion of *MCP1* severely impaired the mitophagy pathway of the fungus under the non-fermentable condition.

### Deletion of *MCP1* Has No Impact on Autophagy and the Cvt Pathway (Cytoplasm to Vacuole Targeting) Under Nitrogen Starvation

**FIGURE 6:**
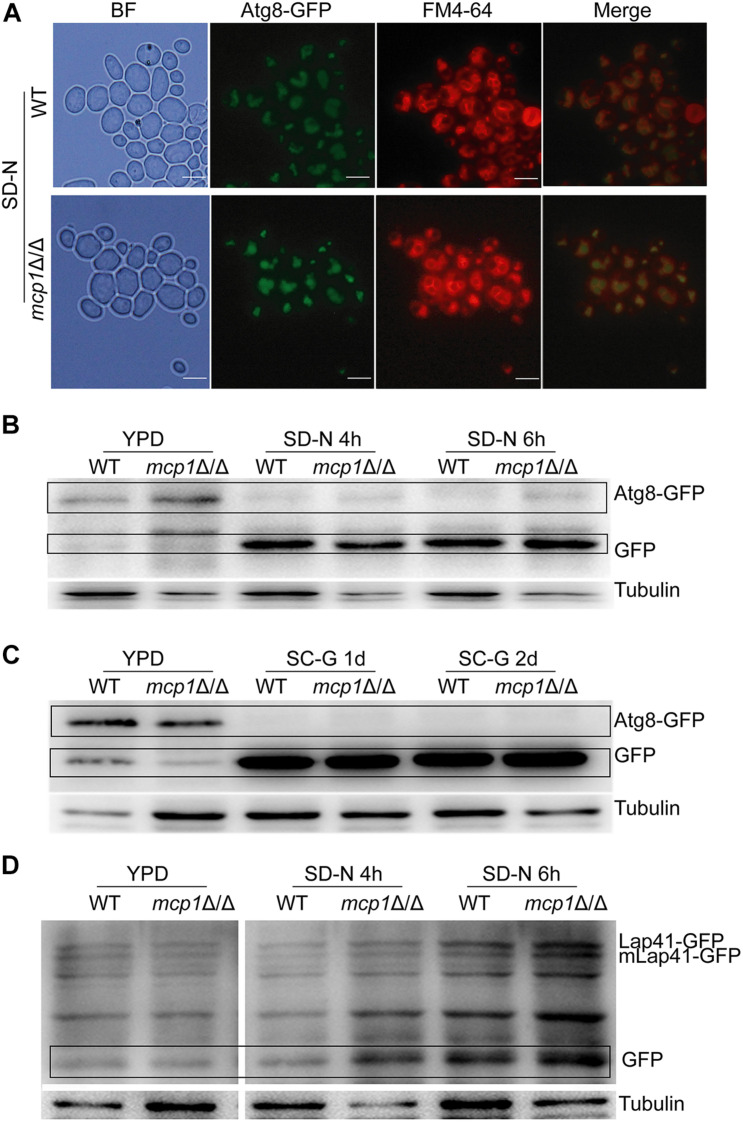
Deletion of *MCP1* does not affect autophagy under nitrogen starvation. **(A)** The influence of Mcp1 on localization of GFP-Atg8 in the SD-N medium. The cells were cultured in SD-N medium for 4 h. The samples were observed after stained by FM4-64. **(B)** Effect of *MCP1* deletion on degradation of Atg8. After cultured in SD-N, cells were collected to extract intracellular proteins. GFP-Atg8 and GFP were analyzed by immunoblotting probed with anti-GFP and anti-tubulin antibodies. **(C)** Atg8 degradation in SC-G medium. **(D)** Degradation of Lap41. Cells was cultured in SD-N medium, intracellular protein is extracted in the special time. Lap41-GFP and GFP were analyzed by immune blotting probed with anti-GFP and anti-tubulin antibodies.

As a protein of autophagosomes, Atg8 protein was transported into vacuoles and degraded in the vacuolar compartment during the process of autophagy (Maeda et al., 2017). Moreover, Lap41 is another marker protein in the process of autophagy. During the process of autophagy, Lap41 is transported to the vacuole through the Cvt pathway, forming mLap41 (Yu et al., 2015). In *S. cerevisiae*, nitrogen starvation culture can activate macroautophagy (Kohda et al., 2007). To explore the role of Mcp1 in the autophagic processes of *C. albicans*, Atg8 and Lap41 were tagged with GFP, respectively, in WT and *mcp1*Δ/Δ mutant (Johansen and Lamark, 2011; Cui et al., 2019). The results confirm that Atg8-GFP is located in the vacuole in SD-N medium (Figure 6A). Furthermore, the degradation of Atg8-GFP and Lap41-GFP was found to be normal in *mcp1*Δ/Δ mutant by Western blotting (Figures 6B–D and Supplementary Figures S4–S9). All the results indicate that macroautophagy and Cvt pathway are independent of Mcp1.

### Deletion of *MCP1* Affected the Vacuolar Functions

**FIGURE 7:**
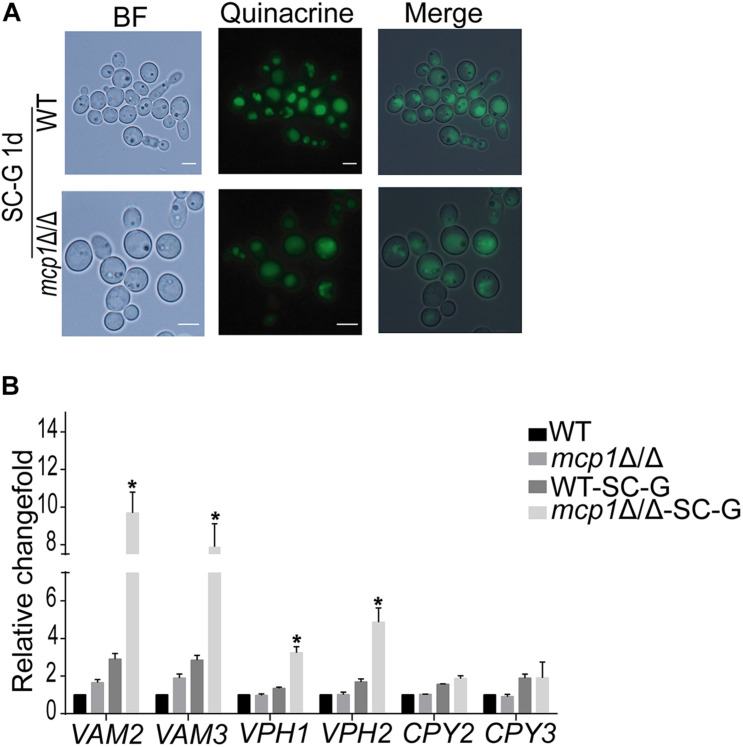
The vacuolar function in *mcp1*Δ/Δ mutant. **(A)** After cultured in SC-G, cells were collected and were stained by Quinacrine. **(B)** Cells were cultured in SC-G medium, and then RNA was extracted. The expression of *VAM2*, *VAM3*, *VPH1*, and *VPH2* were measured by RT-PCR using *ACT1* as the normalization gene. This value represents the mean ± SD with three replicates. *Indicates a significant difference between the mutant and the control strains (*P* < 0.05).

Maintenance of the vacuolar functions is essential for the autophagy process (Klionsky and Ohsumi, 1999). Quinacrine staining revealed that the vacuolar pH is stable in *mcp1*Δ/Δ mutant (Figure 7A). However, the expression of V-ATPase related gene (e.g., *VAM2*, *VAM3*, *VPH1* and *VPH2*) (Jia et al., 2018) and vacuolar hydrolase related gene (*CPY2* and *CPY3*) was significantly up-regulated in *mcp1*Δ/Δ mutant (Figure 7B; Parzych et al., 2018). This study indicates that deletion of *MCP1* affected the activity of V-ATPase.

### Deletion of *MCP1* Impairs Filamentous Growth in *C. albicans*

**FIGURE 8:**
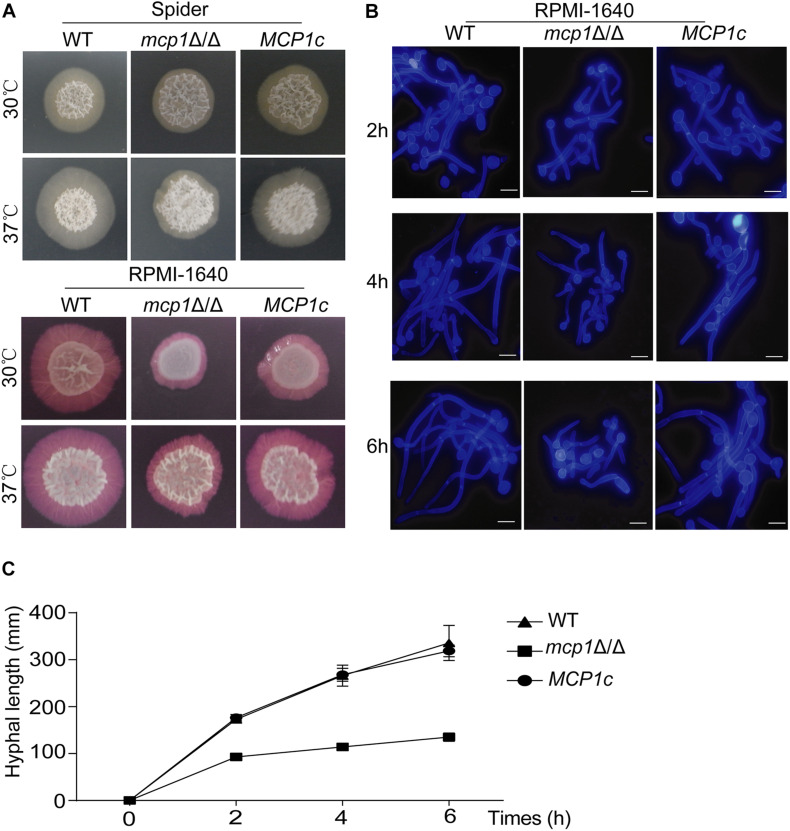
Hyphal growth is impaired in the *mcp1*Δ/Δ mutant. **(A)** Hyphal growth in the solid hypha-inducing media. The cells were spotted onto the indicated hypha-inducing agar plates, and cultured at 30 or 37°C for 5∼7 days before photographed. **(B)** Cells were incubated in liquid RPMI-1640 medium at 37°C and the cells were collected at indicated time points, stained by CFW, and visualized by fluorescence microscopy. **(C)** Determination the growth of hyphal. Strains were cultured in the liquid RPMI-1640 medium. The length of the hypha is measured by Image J.

Some reports indicate that the stability of mitochondria is essential for hypha formation (Potapova et al., 2013). By culturing strains in solid hypha-inducing media (i.e., the Spider and RPMI-1640 medium), it can be seen that deletion of *MCP1* affected the mycelial development of *C. albicans* (Figure 8A; Jia et al., 2018). To further investigate the role of Mcp1 in hyphal development, the strains were cultured in liquid hypha-inducing medium (i.e., the liquid RPMI-1640 medium), and then stained by Calcofluor White (CFW). Also, the growth rate of hyphae of *mcp1*Δ/Δ mutant was significantly lower than WT and *MCP1*c strain (Figures 8B,C). These results revealed that Mcp1 is important for hyphal development in *C. albicans.*

### Deletion of *MCP1* Strongly Impairs Virulence of *C. albicans*

**FIGURE 9:**
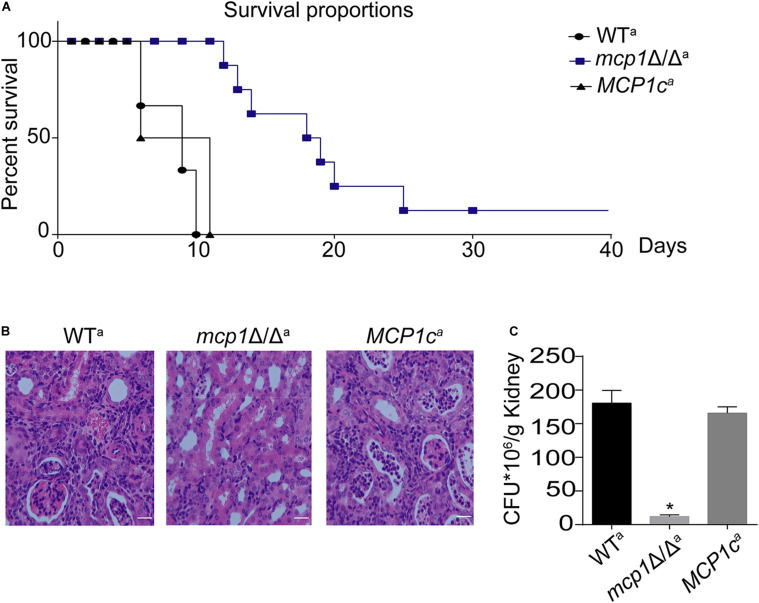
Deletion of *MCP1* significantly attenuates the virulence *of C. albicans*. **(A)** Survival curves of the mice infected by WT, *mcp1*Δ/Δ or *MCP1*c. **(B)** Histological analysis of kidneys. Bar = 50 μm. **(C)** Kidney fungal burden. Values represent mean ± SD. *Indicates significant difference between the groups (*P* < 0.05).

The above studies revealed that deletion of *MCP1* can lead to dysfunction of mitophagy and impair mitochondrial function of *C. albicans*, which are closely related to virulence of *C. albicans*. Hence, it can be speculated that deletion of *MCP1* may reduce the virulence of *C. albicans*. In a mouse model of systemic infection, while all mice were killed by the control strains in 11 days, about all of the mice survived in these days, and 20% mice remained alive even after 30 days (Figure 9A). Furthermore, deletion of *MCP1* led to a significant decrease in kidney fungal burden (Figure 9B), with 10 times lower of kidney CFUs in the mutant-infected mice than that in the control strain-infected mice (Figure 9C). These results indicate that deletion of *MCP1* significantly impaired the virulence of *C. albicans*.

## Discussion

*C. albicans* is a common opportunistic pathogenic fungus in human bodies (Kim and Sudbery, 2011). Some studies have shown that the functions of vacuoles and mitochondria are important to the autophagy process (Noda et al., 2002; AhYoung et al., 2015). This article identifies and names Mcp1, the homolog of ScMcp1 in *C. albicans* (John Peter et al., 2017). Based on fluorescence microscopy techniques and immunotransmission electron microscope (IEM), it can be found that the Mcp1 protein is located both on the mitochondrial and vacuolar membrane. In recent years, some studies have revealed that the vCLAMP is the bypass of mitochondrial-endoplasmic reticulum (ERMES). Moreover, loss of ERMES can lead to abnormal mitophagy in *S. cerevisiae* (Böckler and Westermann, 2014), while ERMES contact is important for autophagy in mammalian (Dikic and Elazar, 2018). However, the mechanism of vCLAMP involvement in autophagy has not been elucidated.

Numerous studies have shown that nitrogen starvation can activate macroautophagy and Cvt (cytoplasm to vacuole targeting) pathway of fungal cells (Cui et al., 2019). Meanwhile, non-fermentable carbon sources can activate the mitophagy (Böckler and Westermann, 2014), which can degrade the excess mitochondria to maintain the development of the cell. In this research, the degradation of Atg8-GFP and Lap41-GFP was normal in *mcp1*Δ/Δ mutant in SD-N medium. However, localization and degradation of Csp37-GFP were evidently blocked in *mcp1*Δ/Δ mutant in SC-G medium. TEM analysis showed that although mitochondria swelled significantly, vesicle packages in *mcp1*Δ/Δ mutant could not form as well. This indicated that Mcp1 may participate in mitophagy, which may be due to the lack of vCALMP protein Mcp1 affecting the formation of mitochondrial autophagosomes. Moreover, *mcp1*Δ/Δ mutant exhibited greater impairments than WT on SC-G media. The death of cells may be closely related to abnormal ROS level or incomplete mitophagy. These outcomes illustrate that Mcp1 participates in the process of mitophagy.

In this research, Mcp1 existed both on the membrane of mitochondria and vacuoles. Therefore, further studies on the function of vacuole have been made (John Peter et al., 2017). The vacuolar morphology, pH, expression of vacuolar hydrolase related gene were normal. However, the expression of V-ATPase related gene was significantly increased. This indicated that deletion of Mcp1 may affect the activity of V-ATPase. Some reports, however, pointed out that the deletion of ScVam6/Vps39p, core protein of vCLAMP, affects the morphology and function of vacuole in yeast (Nakamura et al., 1997; Caplan et al., 2001). This may be due to ScVam6/Vps39p being a necessary gene for vacuolar membrane fusion. Furthermore, the mtDNA quality decreased significantly in *mcp1*Δ/Δ mutant in SC-G media, whereas the amount of mitochondria and production of ATP decreased greatly. All the outcomes prove that when autophagy is activated, mitochondria need to produce more ATP to degrade the damaged mitochondria (Gomes et al., 2011), which gives support to the conclusion that Mcp1 is an important protein in the process of mitophagy. In addition, data in recent years show that mitophagy, as a specific selection process, is precisely regulated by various factors, and is an important regulatory mechanism for cells to remove damaged mitochondria and maintain their own homeostasis. Autophagy related molecules, such as “core” Atg complex, mitochondrial outer membrane molecules Atg32, Atg33, Uth1, and Aup1 in yeast, mammalian cell mitochondrial outer membrane proteins PTEN induced putative kinase 1 (PINK1) and cytoplasmic Parkin (Lemasters, 2005; Böckler and Westermann, 2014), etc., all arise in mitochondrial autophagy. Relevant studies have shown that starvation can induce selective mitochondrial autophagy (Böckler and Westermann, 2014). Under starvation conditions, the ATP production capacity was decreased and the level of ROS was significant increase in cell. ROS causes mitochondrial damage and decreased mitochondrial membrane potential (MMP), thereby inducing mitochondrial autophagy (Dong et al., 2015). In this study, the MMP basically decreased in *mcp1*Δ/Δ mutant, which may be due to the increased mitochondrial damage and ROS level. Further investigation showed that damaged mitochondria could not form autophagosomes, and the expression levels of autophagy-related genes *ATG1*, *ATG5*, and *ATG9* were significantly increased (results not shown) in *mcp1*Δ/Δ mutant. This phenomenon is consistent with the perspective that mitophagy is a targeted defense against oxidative stress, mitochondrial dysfunction and aging (Lemasters, 2005). The above findings confirm that deletion of *MCP1* damage the signal pathway of ROS, stimulates the decrease of MMP and high level of *ATG1*, which induces damaged mitochondria to form autophagosomes under starvation conditions.

The ability of morphological transformation between yeast and hypha is crucial to the pathogenicity of *C. albicans* (Alvarez and Konopka, 2007). This study demonstrates that autophagy is involved in the development and differentiation of fungal cells and the loss of autophagy genes can lead to change of morphology transformation (Cui et al., 2019). The aforementioned findings support that Mcp1 is indispensable to mitophagy. Hence, it can be speculated that the loss of *MCP1* may impair the virulence of *C. albicans*. The hyphae development ability of the *mcp1*Δ/Δ mutant was significantly reduced in both solid and liquid hypha-inducing media. This may be related to the mitochondrial dysfunction caused by the lack of vCLAMP core protein Mcp1 under nutrient deficiency. In addition, the mortality rate of mice infected by the *mcp1*Δ/Δ mutant was reduced, whose kidney fungal load and the ability to prevent invasion were significantly reduced. On the one hand, this may be caused by the deficient ability of filamentous development; on the other hand, the deletion of *MCP1* leading to abnormal mitophagy process causes cell death under environmental pressure generated by host cells. These outcomes manifest that Mcp1 is a potential virulence factor.

In conclusion, this research reveals a critical role of the vCLAMP protein Mcp1 in mitophagy of *C. albicans*, and further confirms the relationship between mitophagy and maintenance of mitochondrial function. The findings will enrich the knowledge of *C. albicans* autophagy and provide new ideas for treatment of *C. albicans* infection.

## Data Availability Statement

The original contributions presented in the study are included in the article/Supplementary Material, further inquiries can be directed to the corresponding author/s.

## Ethics Statement

The animal study was reviewed and approved by the Ethics Committee of Nankai University (20160004).

## Author Contributions

XM conceived, designed the experiments, and wrote the manuscript. YF, JW, DC, DY, and QY performed the experiments. LY analyzed the data. All authors contributed to the article and approved the submitted version.

## Conflict of Interest

The authors declare that the research was conducted in the absence of any commercial or financial relationships that could be construed as a potential conflict of interest.

## Footnotes

1 https://www.ncbi.nlm.nih.gov/Structure/cdd/docs/cdd_search.html
