# Supplementary material for: The Vacuole and Mitochondria Patch (vCLAMP) Protein Mcp1 Is Involved in Maintenance of Mitochondrial Function and Mitophagy in Candida albicans
Source: Front Microbiol. 2021 Feb 4;12:633380. doi: 10.3389/fmicb.2021.633380 (PMC7902011; doi:10.3389/fmicb.2021.633380)
Supplement: Supplementary file 1 [file Data_Sheet_1.PDF]

Supplementary Material

1 Supplementary Figures

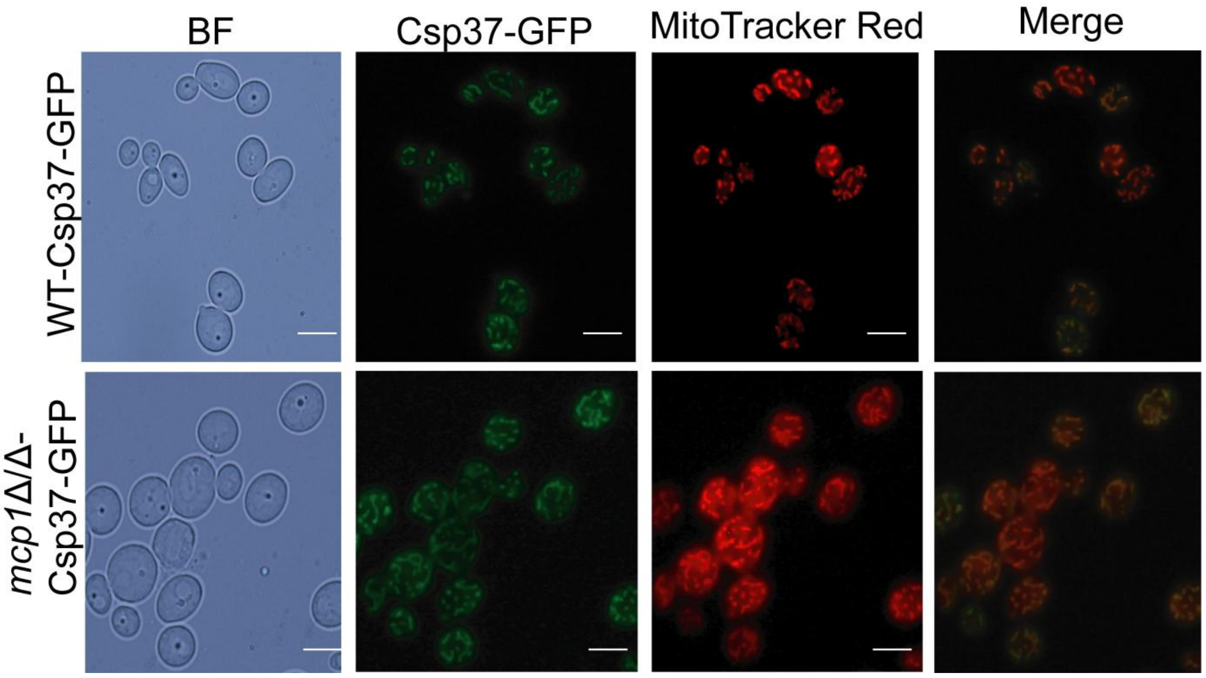

Supplementary Figure 1. Localization of Csp37 was observer in WT-*CSP37-GFP* and *mcp1*Δ/Δ-*CSP37-GFP*. Csp37-GFP: Csp37 localization, green fluorescence. Mito Tracker Red: Mitochondrial localization, red fluorescence. BF: Bright field.

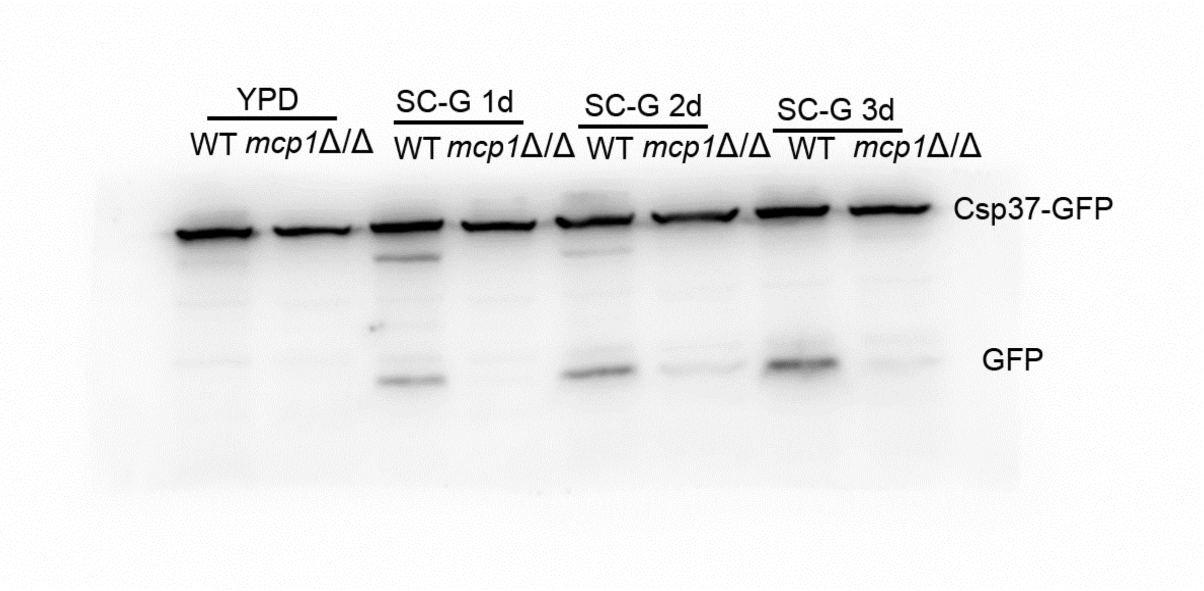

Supplementary Figure 2. A full scan of Figure 4B（Atg8-GFP）entire original gel.

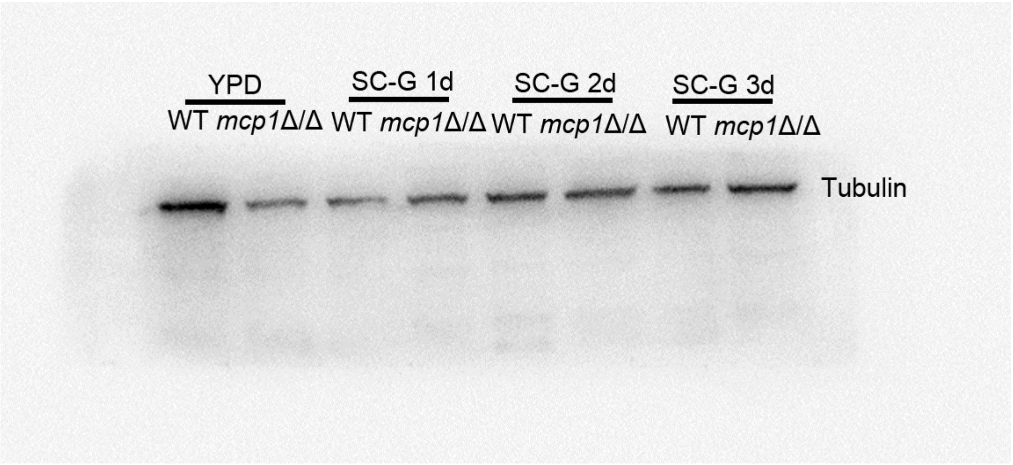

Supplementary Figure 3. A full scan of Figure 4B（Tubulin） entire original gel.

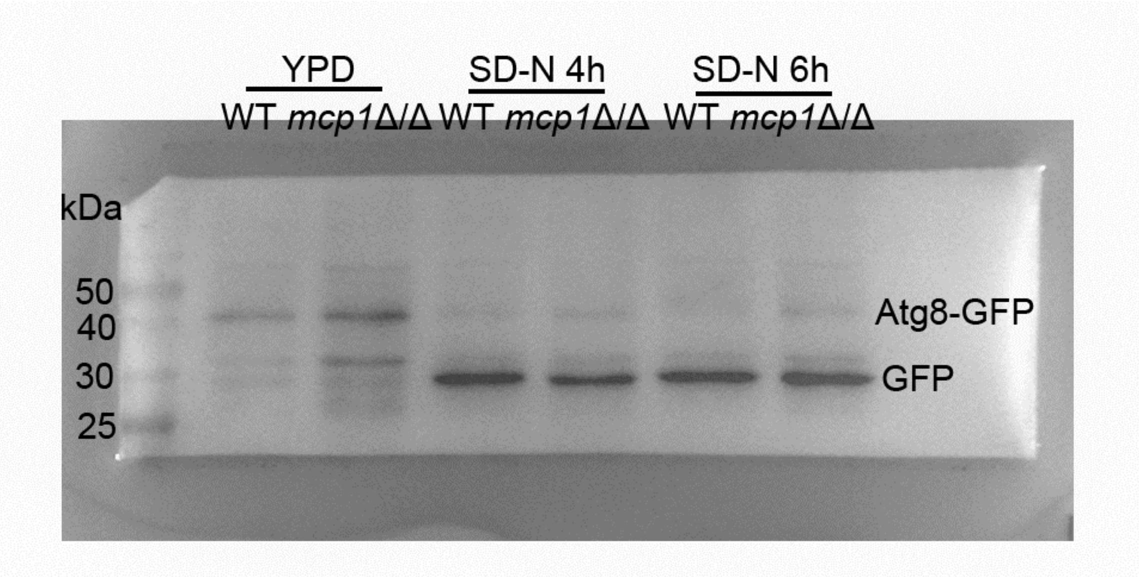

Supplementary Figure 4. A full scan of Figure 6B（Atg8-GFP） entire original gel.

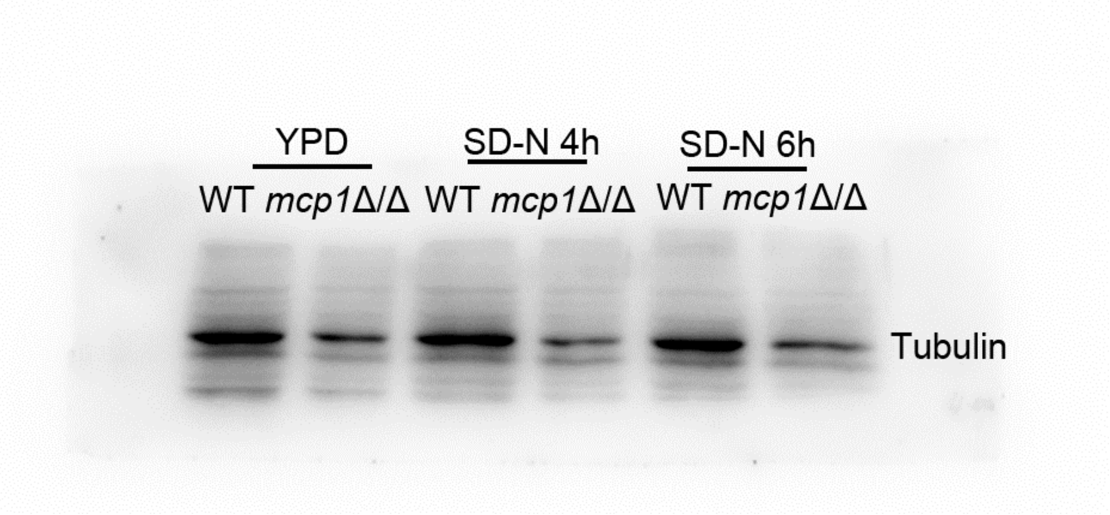

Supplementary Figure 5. A full scan of Figure 6B（Tubulin） entire original gel.

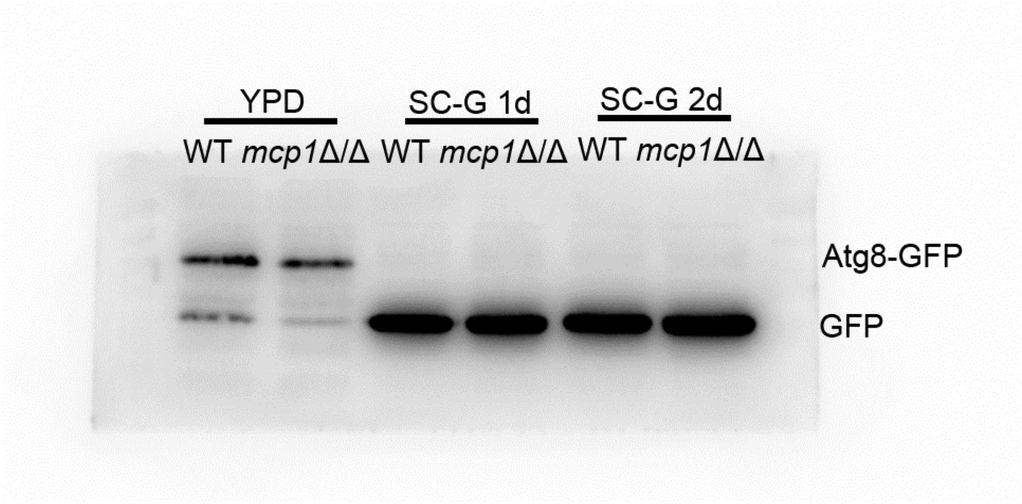

Supplementary Figure 6. A full scan of Figure 6C（Atg8-GFP） entire original gel.

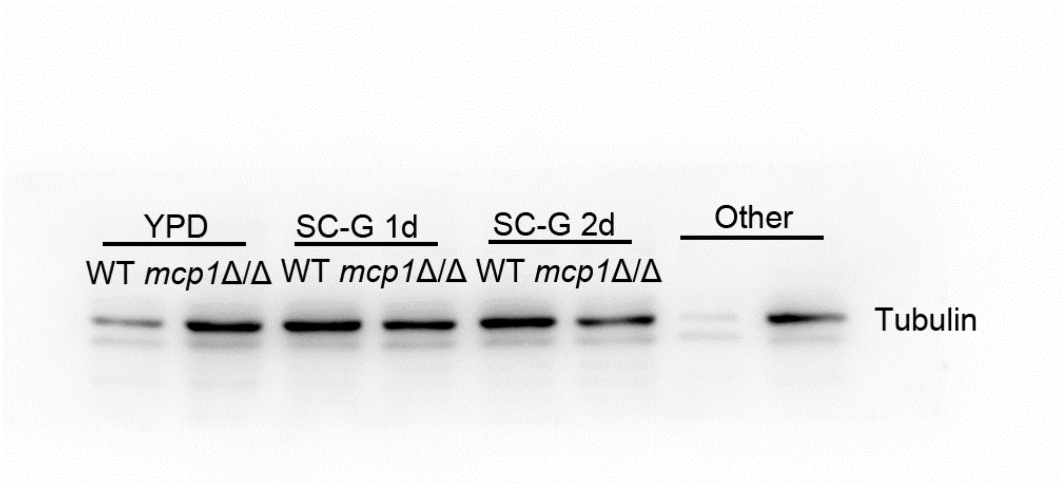

Supplementary Figure 7. A full scan of Figure 6C（Tubulin） entire original gel.

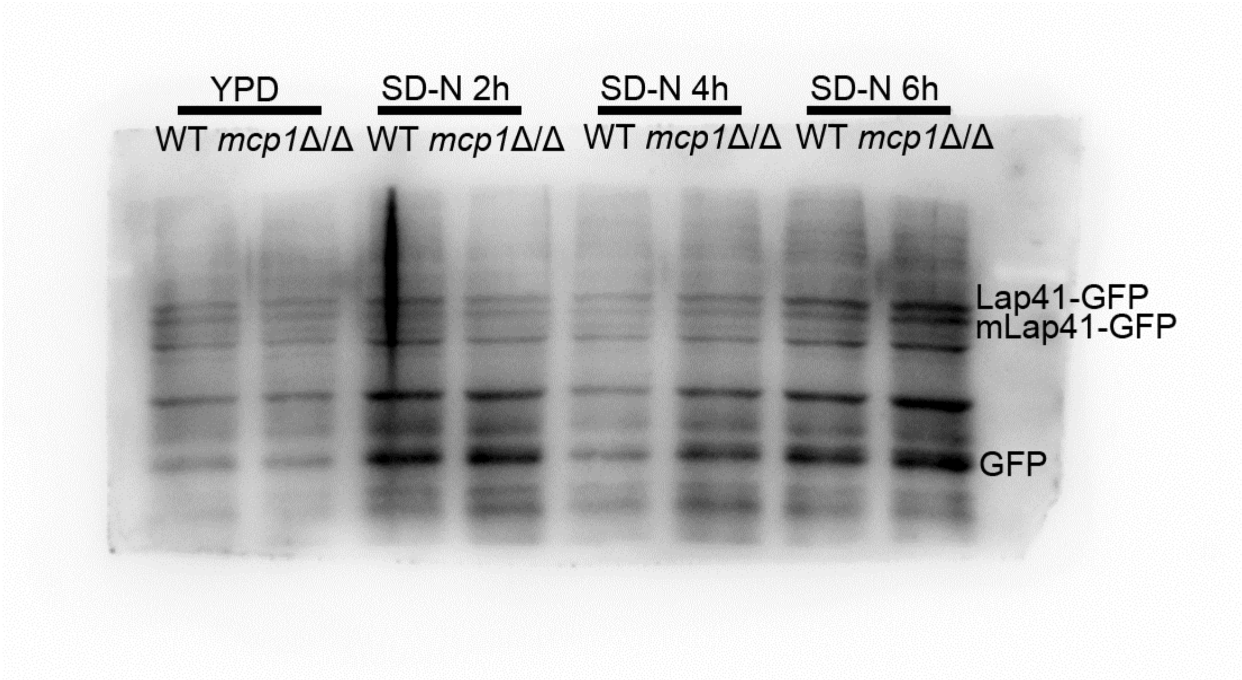

Supplementary Figure 8. A full scan of Figure 6D（Lap41-GFP） entire original gel. We believe that the result of line 3-4 does not affect our conclusion.

.

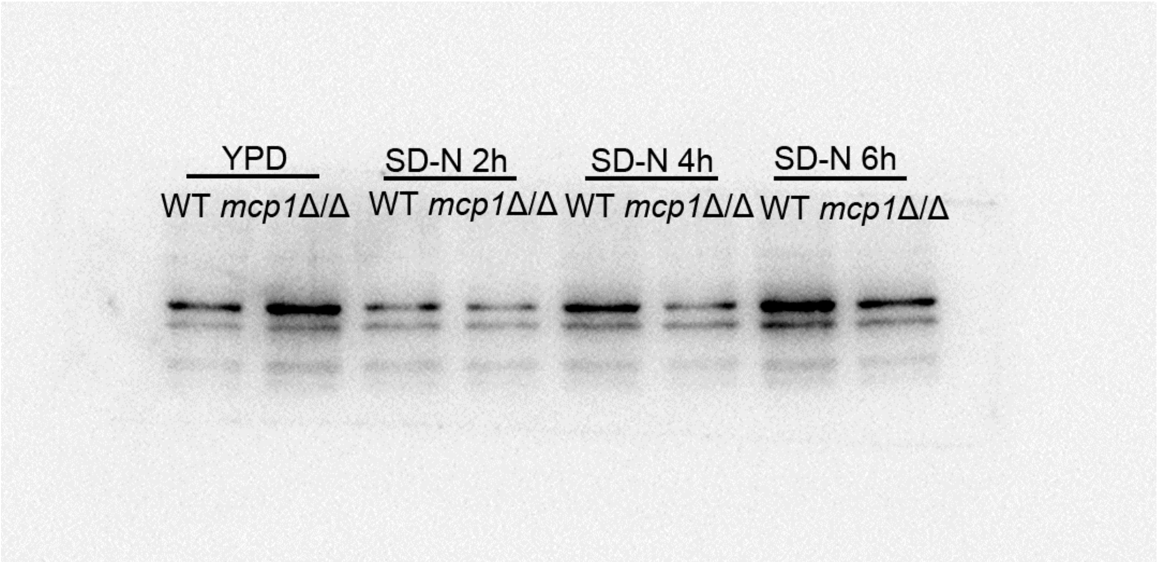

Supplementary Figure 9. A full scan of Figure 6D（Tubulin） entire original gel.

2 Supplementary Tables

Supplementary Table 1 Strains and plasmids used in this study.

Strain/ Plasmid	Description	Source/Purpose
Strains		
BWP17	*ura3Δ::limm434/ura3Δ::limm434 his1::hisG/his1::hisG arg4::hisG/arg4::hisG*	Wilson, et al. (1999)
BWP17[a]	*URA3/ura3Δ::limm434his1::hisG/his1::hisG arg4::hisG/arg4::hisG*	This study
*mcp1*Δ/Δ (+*URA3*)	*ura3Δ::limm434/ura3Δ::limm434his1::hisG/ his1::hisG/arg4::hisG/arg4::hisG mcp1::ARG4/mcp1::dpl200-URA3*	This study
*mcp1*Δ/Δ	*ura3Δ::limm434/ura3Δ::limm434 his1::hisG/his1::hisG arg4::hisG/arg4::hisG mcp1::ARG4/mcp1::dpl200*	This study
*mcp1*Δ/Δ[a]	*URA3/ura3Δ::limm434his1::hisG/his1::hisG arg4::hisG/arg4::hisG mcp1::ARG4/mcp1::dpl200*	This study
MCP1c	*ura3Δ::limm434/ura3Δ::limm434 his1::hisG/his1::hisG arg4::hisG/arg4::hisG mcp1::ARG4/mcp1::dpl200,* pDDB78-*MCP1*	This study
MCP1c[a]	*URA3/ura3Δ::limm434his1::hisG/his1::hisG arg4::hisG/arg4::hisG mcp1::ARG4/mcp1::dpl200,* pDDB78-*MCP1*	This study
*mcp1*Δ/Δ-*CSP37-GFP*	*ura3Δ::limm434/ura3Δ::limm434 his1::hisG/his1::hisG arg4::hisG/arg4::hisG mcp1::ARG4/ mcp1::dpl200 CSP37/CSP37-GFP::URA3*	This study
WT-*ATG8-GFP*	*ura3Δ::limm434/ura3Δ::limm434 his1::hisG/his1::hisG arg4::hisG/arg4::hisG ATG8/ATG8-GFP::URA3*	This study
*mcp1*Δ/Δ-*ATG8-GFP*	*ura3Δ::limm434/ura3Δ::limm434 his1::hisG/his1::hisG arg4::hisG/arg4::hisG mcp1::ARG4/ mcp1::dpl200 ATG8/ATG8-GFP::URA3*	This study
WT-*LAP41-GFP*	*ura3Δ::limm434/ura3Δ::limm434 his1::hisG/his1::hisG arg4::hisG/arg4::hisG LAP41/LAP41-GFP::URA3*	This study
*mcp1*Δ/Δ-*LAP41-GFP*	*ura3Δ::limm434/ura3Δ::limm434 his1::hisG/his1::hisG arg4::hisG/arg4::hisG mcp1::ARG4/ mcp1::dpl200 LAP41/LAP41-GFP::URA3*	This study
WT-*CSP37-GFP*	*ura3Δ::limm434/ura3Δ::limm434 his1::hisG/his1::hisG arg4::hisG/arg4::hisG*	This study

*mcp1*Δ/Δ-*CSP37-GFP*	*CSP37/CSP37-GFP::URA3 ura3Δ::limm434/ura3Δ::limm434 his1::hisG/his1::hisG arg4::hisG/arg4::hisG mcp1::ARG4/ mcp1::dpl200 CSP37/CSP37-GFP::URA3*	This study
pRS-ArgΔ*Spe*I	Amp^r *ARG4*	Wilson, et al. (1999)
pLUBP	Amp^r *IRO1-URA3*	Gerald Fink (2003)
pDDB78-*MCP1*	Amp^r *MCP1 TRP1 HIS1*	This study
pAu34M-GFP	Amp^r P_{ACT1}*-GFP URA3*	Yu et al. (2014)
pAu34M–*MCP1-GFP*	Amp^r P_{ACT1}*-MCP1-GFP URA3*	This study

[a] Strain that the *URA3* gene was reintroduced at its common locus.

Supplementary Table 2 Primers used in this study.

Primer	Sequence (5'→3')	Function
MCP1-5DR	TGTGTTGTCCTGATTTATCTCTTGGTT TTTGGGTTTTTTTTTTTTTCGGTTGTTC GTGGGTTTCCCAGTCACGACGTT	Gene knockout
MCP1-3DR	TGCACTCAGGTACTACCTCTATCAATA TTGGCAACCACCTTTCTCTTTATTTTA CCACACTGGAATTGTGAGCGGATA	Gene knockout
MCP1-5det	GCTGTTGTTGTTGAAATTGG	Genetic validation
MCP1-3det	ATACTAGACCCGATGTGACT	Genetic validation
MCP1-5com	ATAAGAATGCGGCCGCCCAACCTAAT CCATAGCCAT	Complement gene
MCP1-3com	GGGGTACCAGTGTGGTCGTTGTTGTT AC	Complement gene
MCP1-5GFP	CCGCTCGAGATGTCACAAGAAGATTT GCTAAC	Transcriptional fusion
MCP1-3GFP	TCCCCCGGGCAACCAGAACGACCTTA AGTA	Transcriptional fusion
COX2-5RT	TAGATGTACCTACACCTTGA	RT-PCR
COX2-3RT	CCTATAGAGTCAACAAAATC	RT-PCR
NAD2-5RT	ACATACATAGTTTATATCGC	RT-PCR

NAD2-3RT	GAGAGTAAGATTGTAATTCA	RT-PCR
NAD5-5RT	TACTTGAATTAGTCTAGGTG	RT-PCR
NAD5-3RT	CTACAAACTCTCAACCAATG	RT-PCR
ATP6-5RT	TTCACCTTTAGATCAATTCG	RT-PCR
ATP6-3RT	AAACTAAATGATACAATAGC	RT-PCR
URA3-5inner	CGCGGGATTTGGATGGTAT	URA test
URA3-3inner	TCTTGGCTCTTGGTTGGTG	URA test
ACT1-5RT	GGTAGACCAAGACATCAAGG	RT-PCR
ACT1-3RT	CCGTGTTCAATTGGGTATCT	RT-PCR
LAP41-GFP-1	AATTCTTCTATGGTTTCTTCAAGAATT GGAGAGATGTCTATGATAATTTTGTT GATTTAGGTGGTGGTTCTAAAGGTGA AGAATTATT	*GFP* localization for *LAP41*
LAP41-GFP-2	ACTAACAAATAACTAATACTTGCAAA TCAACTTTTAATGATTCCTTTTCCTGT TGTTCAATGTTCGACTAGAAGGACCA CCTTTGATTG	*GFP* localization for *LAP41*
CSP37-GFP-1	TTGGTGATGACGTCTACAATGATTTTG CTAAAAGATTTGACGATTTGTTTAACC GTAAAGGTGGTGGTTCTAAAGGTGAA GAATTATT	*GFP* localization for *CSP37*
CSP37-GFP-2	AATATAAGAATATTACGTCTCTTTTCT TTTCTTTTTCTGTTTATGCTAACATCAT TTTTAAAAAAACCTAGAAGGACCACC TTTGATTG	*GFP* localization for *CSP37*
VAM2-5RT	AAGAAGAGGAAGAGCCACC	RT-PCR
VAM2-3RT	AAACTGATAGCACGGACGC	RT-PCR
VAM3-5RT	CACAAGTTGACCAAGACC	RT-PCR
VAM3-3RT	CTCGTTAGATGCCTGTTG	RT-PCR
VPH1-5RT	AGTGATGAGGAGGAGCAGGA	RT-PCR
VPH1-3RT	TGCCAAGGAAAGAGCCCAT	RT-PCR
VPH2-5RT	GCCAGGAACAAGAATACAG	RT-PCR
VPH2-3RT	ACAACCACTTCAGCAACC	RT-PCR
CPY2-5RT	AACCAATGGGCTGTGGTGAA	RT-PCR
CPY2-3RT	TGCTCGGCACAATCCTTTCT	RT-PCR
CPY3-5RT	TGGCTTAATGGTGGTCCTGG	RT-PCR
CPY3-3RT	AGTCCTGTGAAGCTTGGTCG	RT-PCR
